# Angiopoietin-Like Protein 3 Promotes Preservation of Stemness during *Ex Vivo* Expansion of Murine Hematopoietic Stem Cells

**DOI:** 10.1371/journal.pone.0105642

**Published:** 2014-08-29

**Authors:** Elnaz Farahbakhshian, Monique M. Verstegen, Trudi P. Visser, Sima Kheradmandkia, Dirk Geerts, Shazia Arshad, Noveen Riaz, Frank Grosveld, Niek P. van Til, Jules P. P. Meijerink

**Affiliations:** 1 The Department of Hematology, Erasmus Medical Center, Rotterdam, the Netherlands; 2 The Department of Pediatric Oncology/Hematology, Erasmus Medical Center, Rotterdam, the Netherlands; 3 The Department of Surgery, Erasmus Medical Center, Rotterdam, the Netherlands; 4 The Department of Cell Biology, Erasmus Medical Center, Rotterdam, the Netherlands; Emory University, United States of America

## Abstract

Allogeneic hematopoietic stem cell (HSC) transplantations from umbilical cord blood or autologous HSCs for gene therapy purposes are hampered by limited number of stem cells. To test the ability to expand HSCs *in vitro* prior to transplantation, two growth factor cocktails containing stem cell factor, thrombopoietin, fms-related tyrosine kinase-3 ligand (STF) or stem cell factor, thrombopoietin, insulin-like growth factor-2, fibroblast growth factor-1 (STIF) either with or without the addition of angiopoietin-like protein-3 (Angptl3) were used. Culturing HSCs in STF and STIF media for 7 days expanded long-term repopulating stem cells content *in vivo* by ∼6-fold and ∼10-fold compared to freshly isolated stem cells. Addition of Angptl3 resulted in increased expansion of these populations by ∼17-fold and ∼32-fold, respectively, and was further supported by enforced expression of Angptl3 in HSCs through lentiviral transduction that also promoted HSC expansion. As expansion of highly purified lineage-negative, Sca-1^+^, c-Kit^+^ HSCs was less efficient than less pure lineage-negative HSCs, Angptl3 may have a direct effect on HCS but also an indirect effect on accessory cells that support HSC expansion. No evidence for leukemia or toxicity was found during long-term follow up of mice transplanted with *ex vivo* expanded HSCs or manipulated HSC populations that expressed Angptl3. We conclude that the cytokine combinations used in this study to expand HSCs *ex vivo* enhances the engraftment *in vivo*. This has important implications for allogeneic umbilical cord-blood derived HSC transplantations and autologous HSC applications including gene therapy.

## Introduction

Hematopoietic stem cells (HSCs) have the ability to self-renew and to give rise to cells of all blood lineages. This makes HSCs a valuable source for treatment of patients with genetic blood disorders through cell- or gene-based therapies [1]–[4]. These therapies are restricted by the limited availability of suitable, human leukocyte antigen (HLA)-matched donors. Additionally, if autologous cells for genetic modification are concerned, limited numbers of cells can be retrieved per patient. Umbilical cord blood (UBC) potentially provides an alternative and abundant source of donor HSCs, if the number of HSCs could be increased *in vitro* [5], [6]. Optimization of *in vitro* expansion protocols would therefore facilitate successful transplantations using UCB-derived HSCs or genetically-modified autologous HSCs [7], [8].

Early attempts to expand HSC *in vitro* resulted in a preferential expansion of committed progenitor cells without preserving stemness, resulting in defective long term hematopoiesis [9]. However, the knowledge on hematopoietic stem cell expansion has increased, and new methods for promoting expansion of stem cells while retaining stemness have been developed. Ectopic expression of the transcription factors, such as homeobox B4 (HoxB4) or apoptotic regulators such as Bcl-2 have been investigated and can result in robust HSC expansion [10], [11]. However, the long term consequences of constitutive activation of anti-apoptotic pathways triggered by specific factors such as Bcl-2 or HoxB4 is not yet fully investigated. Another obstacle is the delivery of these proteins, which may require vector-based vehicles, which should be efficient and not genotoxic [12]–[15]. To circumvent these problems, it would therefore be preferable to develop methodology to expand HSC without the introduction of foreign DNA sequences.

Several growth factors have been identified over the years that enhance the self-renewal capacity of mouse HSCs, including ligands for various pathways such as Notch1 [16], stem cell factor (SCF) [17], thrombopoietin (TPO) [18], [19], fms-like tyrosine kinase-ligand (Flt3-L) [20], fibroblast growth factor (FGF-1) [21], [22] or WNT-pathway factors like Wnt3a [23]. The Lodish group identified a fetal liver stromal cell population that produces high levels of insulin growth factor-2 (IGF-2) and angiopoietin-like proteins in addition to SCF and delta-like NOTCH1 ligands. These factors were shown to support HSC expansion *in vivo* [24]–[26]. The combination of IGF-2, angiopoietin-like 2 (Angptl2), and angiopoietin-like 3 (Angptl3) growth factors also support HSC expansion *in vitro* [25], [26]. Various studies support a pivotal role for Angptl3 in regulating HSC self-renewal capacity [26]–[29]. This was confirmed by results from the Angptl3 knock-out mouse model that demonstrate reduced numbers of quiescent HSCs as well as reduced repopulation capacity in transplantation experiments [27]. Angptl3 is expressed by endothelial and other stromal cells in the bone marrow and binds as an extrinsic factor to receptors on HSCs [27]. At present, the receptor for Angptl3 is not clear as the immune-inhibitory receptor human leukocyte immunoglobulin-like receptor B2 (LILRB2) and the mouse orthologue paired immunoglobulin-like receptor (PIRB) have been identified as receptors for several angiopoietin-like proteins (Angptls) including Angptl2, −5, and −7, but this is unclear for Angptl3 [28]. Binding of Angptls to its receptor results in reduced expression of Ikaros and activate self-renewal capacity [27]. Overexpression of Ikaros in HSCs was shown to diminish repopulation capacity [27]. The combination of saturated levels of SCF, TPO, IGF2, FGF1 and Angptl3 has been proven as a suitable cocktail that promotes expansion of long-term repopulating HSCs (LT-HSCs) numbers up to ∼30-fold [26].

In the present study, we tested the preservation and expansion of long- and short-term HSCs *in vitro* in serum-free culture conditions in the presence of SCF, TPO, IGF2 and FGF-1 (STIF) [26] or SCF, TPO and FLT3-L (STF) [9]. Long-term repopulating capacity was investigated for cultured HSCs under various conditions followed by transplantation into sub lethally irradiated mice. We investigated a potential additive effect of mAngptl3, that may exert a direct effect on HSCs. We also tested the potential leukemogenic or toxic effects of ectopic expression of Angptl3 in transplanted mice.

## Material and Methods

### Mice

Female α-thalassemic BALB/c mice between 8 to 12 weeks of age were used as bone marrow (BM) recipients, and healthy male littermates were used as donors for HSCs. Mice were bred and housed under specific pathogen free (SPF) conditions at the Experimental Animal Facility of Erasmus Medical Center (Rotterdam, the Netherlands). All experiments have been approved by the local ethical committee for animal experiments and are in accordance with national legislation.

### Stem cell isolation

Lineage negative (Lin^−^) cells were purified from BM using the BD IMag Mouse Hematopoietic Progenitor Cell Enrichment Set (BD Biosciences, Breda, The Netherlands) according to the manufacturer's instructions. HSC were further enriched from the Lin^−^ cell population by sorting Sca-1^+^/c-kit^+^ (LSK) cell populations using a BD FACS Aria flow cytometry (BD Biosciences). For this, Lin^−^ cells were incubated with c-kit–allophycocyanin (APC; BD Biosciences) and Sca-1–R-phycoerythrin (PE; BD Biosciences), and washed once with Hank's solution supplemented with HEPES (300 mOsm) prior to sorting.

### Construction of lentiviral vector plasmids

A codon optimized m-Angptl3 cDNA (Genscript) was developed and excised from plasmid pUC57-m-Angptl3 by SalI and XmaI digestion, and cloned into the third generation pCCLsin-cppt-SV40polyA-eGFP-minCMV-hPGK-WPRE lentiviral (LV-GFP) vector [30] to generate the pCCLsin-cppt-SV40polyA-eGFP-minCMV-hPGK- mAngptl-3-WPRE vector. This bidirectional vector drives expression of both Angptl3 and eGFP, and will be denoted as LV-Angptl3-GFP.

### Production of lentiviral vectors

Lentiviral (LV) particle production was done by transfecting the LV-Angptl3-GFP vector in combination with pMDL-g/pRRE, pMD2-VSVg and pRSV-Rev helper plasmids into HEK 293T cells using standard calcium phosphate as previously described [31], [32]. Lentiviral particles were concentrated through ultracentrifugation for 2 hours at 20,000 rpm and collected at 4°C. The multiplicity of infection (MOI) of LV-Angptl3-GFP or LV-GFP was determined on HeLa cells by serial dilution, and the percentage of eGFP-positive cells was estimated by flow cytometry. Angptl3 protein expression was detected by a standard Western-blot procedure using the mouse monoclonal anti-ANGPTL3 (1D10) antibody (Novus Biologicals, Cambridge, United Kingdom).

### *In vitro* suspension culture

Fresh Lin^−^ or LSK cells were cultured in 24-well plates (Costar tissue-culture treated polystyrene, Corning, Corning, NY, USA) at a density of 4–6×10^4^/ml in enriched serum-free Dulbecco's modified Eagle's medium (DMEM) supplemented with 1% (wt/vol) bovine serum albumin (BSA), 0.3 mg/L human transferrin, 0.1 µM sodium selenite, 1 mg/L nucleosides (cytidine, adenosine, uridine, guanosine,2′-deoxycytidine, 2′-deoxyaenosine, thymidine and 2′-deoxyguanosine; Sigma, St. Louis, MO, USA), 0.1 mM ß-mercaptoethanol, 15 µM linoleic acid, 15 µM cholesterol, 100 U/ml penicillin and 100 µg/ml streptomycin as described previously [33], [34]. The enriched DMEM medium was supplemented with murine SCF (50 ng/ml, R&D, Abingdon, UK), murine TPO (20 ng/ml, R&D), murine IGF2 (20 ng/ml, R&D), human FGF-1 (10 ng/ml, R&D) and heparin (10 µg/ml, Sigma) and will be further denoted as STIF medium [26]. Alternatively, enriched DMEM medium was supplemented with murine SCF (50 ng/ml, R&D), murine TPO (20 ng/ml, R&D) and human FLT3-L (50 ng/ml, R&D) and is further denoted as STF medium [9]. Both STIF and STF media were supplemented with murine Angptl3 (200 ng/ml, R&D) and will be referred to as STFA3 or STIFA3 media, respectively. All cells were maintained at 37°C in a humidified incubator at 10% CO2 levels.

### Lentiviral hematopoietic stem cell transduction and sorting

Lin^−^ donor cells were transduced overnight with LV-Angptl3-GFP or the LV-GFP at a cell density of 106 cells/ml using a MOI of 10. During this transduction procedure, cells were maintained in serum-free DMEM medium that was supplemented with various growth factors including murine SCF (100 ng/ml, R&D), murine TPO (20 ng/ml, R&D) and murine IGF-2 (20 ng/ml, R&D). The following day, cells were diluted to 5×10^4^ cell/ml and cultured for another 24 hrs. Subsequently, Lin^−^ GFP^+^ cells were flow-sorted with a purity of >90%. Sorted cells were used in experiments directly or following preculturing in STIF media. All of the cells were incubated at 10% CO2 levels and 37°C.

### *In vitro* clonogenic progenitor assays

Frequencies of HSCs and progenitor cells were estimated using semi-solid colony assays. For this, freshly sorted or cultured (transduced) Lin^−^ (2×10^3^) or LSK (0.2×10^3^) cells were plated in 35 mm culture dishes (BD BioCoat Collagen IV, tissue-culture treated polystyrene) that contained 1 ml of enriched DMEM culture medium that was supplemented with 0.8% (wt/vol) methylcellulose (Methocel A4M Premium Grade, Dow Chemical, Barendrecht, The Netherlands) as described [33], [35]. All primary data is shown in table S1 and S2.

For colony- forming unit granulocyte-macrophage (CFU-GM) differentiation assays, Lin^−^/LSK cells were cultured in methylcellulose-enriched DMEM medium that was further supplemented with 10 ng/ml mouse interleukin-3 (mIL-3), 100 ng/ml m-SCF and 20 ng/ml granulocyte macrophage colony- stimulating factor (GM-CSF). For burst-forming erythroid unit (BFU-E) assays, Lin^−^/LSK cells were incubated in methylcellulose-enriched DMEM medium that was supplemented with 100 ng/ml m-SCF and 4 U/ml human erythropoietin (H-EPO, Behringwerke, Marburg, Germany). Cells were maintained for 14 days prior to microscopic analysis and the total numbers of colonies were counted. Each experiment was carried out in duplicate.

### Colony forming unit spleen (CFU-S)

Lin^−^ or LSK donor cells were transplanted into lethally irradiated (8 Gy) BALB/c female recipient mice (n = 6–10 per group). A total of 1,000 or 3,000 of freshly isolated Lin^−^ cells were transplanted or equivalents of 100 or 1,000 cells that were cultured for 4 to 7 days in STF, STFA3, STIF or STIFA3 media. For LSK cell transplantation, a total of 30 or 100 freshly isolated LSK cells were transplanted directly or following culture for 7-days in STF, STFA3, STIF or STIFA3 media. For LSK cell transplantations, 1000 irradiated non-selected BM cells (50 Gy) were co-transplanted to improve homing. No splenic colonies were observed in control mice that were transplanted with 1000, 50 Gy- irradiated BM cells only. All primary data is shown in table S1 and S2.

In addition, Lin^−^ cells were transduced with LV-GFP or LV-Angptl3-GFP. The GFP^+^ population was sorted 2 days after transduction, and 1000 Lin^−^ GFP^+^ cells were intravenously transplanted into lethally irradiated (8 Gy) BALB/c recipients (n = 7–10 mice per group). Twelve days after transplantation, mice were sacrificed and the spleens were incubated in fixation buffer (70% ethanol supplemented with 5% acetic acid and 2% formalin). The number of spleen colonies (CFU-S) was counted.

### Long-term repopulation ability assay (LTRA)

Lin^−^ or LSK male donor cells were transplanted into sub-lethally irradiated (6 Gy) female α-thalassemia mice. Blood was collected monthly for six months following transplantation, and blood cell counts were measured with a Vet Animal Blood Counter hematology analyzer (Scil Animal Care Company GmbH). Red blood cell (RBC) chimerism were measured following preparation of mouse peripheral blood in 1 ml 0.6% NaCl buffer, and then populations of microcytic thalassemia RBCs and healthy RBCs were determined by flow cytometry (FACS Calibur, BD Biosciences). Percentage of chimerism was calculated using the following formula: [−0.6+SQUART((0.62–4×0.002×(10.43-donor cells %)))/0.004] [36].

In another experiment, donor Lin^−^ cells were transduced with control LV-GFP or LV-Angptl3-GFP. GFP^+^ cells were sorted 2 days after transduction as described above. Lin^−^, GFP^+^ cells (10,000 cells) were transplanted into sub lethally irradiated (6 Gy) female α-thalassemia recipient mice. Blood was collected at one, four, six and nine months following transplantation to determine the percentages of GFP^+^ peripheral blood cells. Similarly, percentages of GFP^+^ white blood cell types in BM or spleens were measured 9 months after transplantation, using antibodies against Sca-1, c-Kit, CD4, CD8, CD19, and CD11b (Miltenyi Biotec, BD Biosciences).

### Y-chromosome Q-PCR

Y-chromosome Q-PCR was performed to detect the percentage of donor leukocyte chimerism in recipient mice following transplantation. DNA was extracted from BM using a DNeasy Blood & Tissue Kits (Qiagen, Germany). Specific primers for the *Sry* locus in the Y-chromosome were designed using Beacon software (New Orleans, LA, USA): Sense primer, 5′-TCA-TCG-GAG-GGC-TAA-AGT-GTC-AC-3′; antisense primer, 5′-TGG-CAT-GTG-GGT-TCC-TGT-CC-3′. As a control for total DNA, the following *GAPDH* primers were used: sense primer 5′-ACG-GCA-AAT-TCA-ACG-GCA-CAG-3′; antisense primer, 5′-ACA-CCA-GTA-GAC-TCC-ACG-ACA-TAC-3′. Each reaction mixture contained 8 µl of DNA template (70 ng), 8 µl SYBR Green PCR Master Mix (Applied Biosystems, Foster City, CA) and1.25 µl of each primer (5 ng/µl) in a final reaction volume of 25 µl. Q-PCR was performed in a Biorad MyiQ thermocycler as follows: 95°C for 3 min, followed by 40 cycles of 95°C for 15 seconds and 60°C for 45 seconds.

### Statistical analysis

Data are expressed as the mean ± standard deviation (SD). Statistical significance between nominal data point comparisons was determined using the Mann-Whitney-U test. Standard deviations of colony counts were calculated on the assumption that crude colony counts show a normal Poisson distribution.

## Results

### The effect of Angptl3 on *ex vivo* expansion and differentiation capacity of murine hematopoietic stem cells

**Figure 1:**
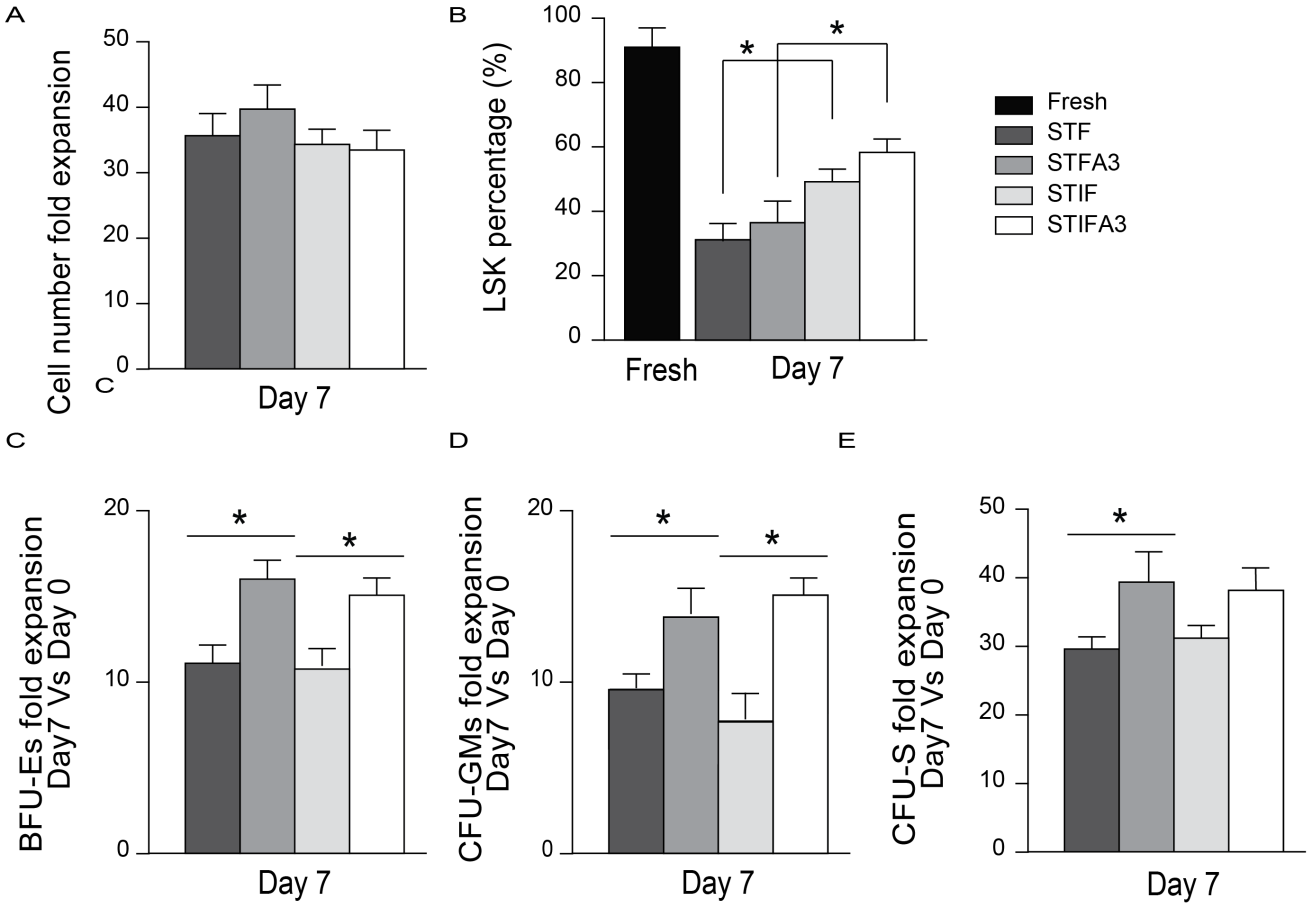
The effect of Angptl3 on progenitors and ST-HSCs in LSK cell populations. Lin^−^ Sca-1^+^ c-kit^+^ (LSK) cells were cultured in STF or STIF media with or without Angptl3 for 7 days. (**A**) The mean fold increase in total cell numbers was measured in comparison to day 0. The results of five independent duplicate experiments are shown. (**B**) LSK cells were cultured for 7 days, and Sca-1 and c-kit markers were determined by flow cytometry. (C) BFU-E and (D) CFU-GM colony forming-units of LSK cells cultured for 7 days are shown relative to fresh LSK cells. The results of five independent experiments are shown. (E) The CFU-S (12-day) fold expansion of LSK cells cultured for 7 days in STF or STIF media with or without Angptl3 relative to fresh LSK cells. For each group, splenic colonies of 6 mice were counted. The results of two independent experiments are shown. Data is presented ± standard deviation (SD), *P≤0.05.

The expansion of murine hematopoietic stem cell (HSC) was tested using various media conditions; Lin^−^ or purified LSK cells were cultured *ex vivo* in media supplemented with STF, STFA3, STIF or STIFA3 growth factor cocktails (see materials and methods). Culturing LSK cells for 7 days in STF- or STIF- media led to ∼35-fold expansion in total cell number (Figure 1A). The LSK phenotype was best preserved in STIF media (at a level of 45±3%) compared to STF media (at 30±4%) (Figure 1B). Addition of mAngptl3 did not result in significant increases in the preservation of LSK phenotype during expansion. The expansion rate of Lin^−^ cells also did not differ between STF or STIF media (Figure S1A). Again, supplementing the media with Angptl3 did not result in a significant increase in total cell numbers. Culturing Lin^−^ cells in STFA3 media for ten days resulted in a near 60-fold expansion of total cell numbers.

We next tested the differentiation capacity of *in vitro* expanded hematopoietic progenitor cells by carrying out various colony-forming unit assays on Lin^−^ cells (Figure S1B-C) or sorted LSK cells cultured in STF, STFA3, STIF or STIFA3 media (Figures 1C and 1D). BFU-Es colony forming units were boosted 11±1 fold for LSK cells cultured in STF and STIF media for 7 days compared to non-cultured LSK cells (Figure 1C). Addition of Angptl3 significantly increased the total number of colony forming units 16±1 and 15±1 fold for STFA3 and STIFA3 media, respectively. For granulocyte- macrophage progenitor cells, culturing in STF or STIF media for 7 days expanded the number of CFU-GM colonies by 9-fold and 7-fold, respectively. Again, culturing with Angptl3-supplemented media further increased the number of CFU-GM colonies to 13±2 and 14±1 fold using STFA3 and STIFA3 media, respectively (Figure 1D). Similar results were obtained from the number of BFU-E and CFU-GM colony forming units in the Lin^−^ subset (Figures S1B–C).The colony forming unit spleen assay (CFU-S) was used to assess the effect of Angptl3 on short-term HSC (ST-HSC) in Lin^−^ and LSK cells (Figure 1E, Figure S1D). LSK cells pretreated in STF or STIF media showed a 28±2 and 30±2 fold increase in splenic colonies, respectively. Culturing LSK cells in STFA3 or STIFA3 media boosted CFU-S colony formation to 38±4 and 37±3-fold relative to control mice. In case of STFA3 the increase was significantly higher relative to STF.

### Effect of Angptl3 on long-term HSCs expansion from LSK cells

**Figure 2:**
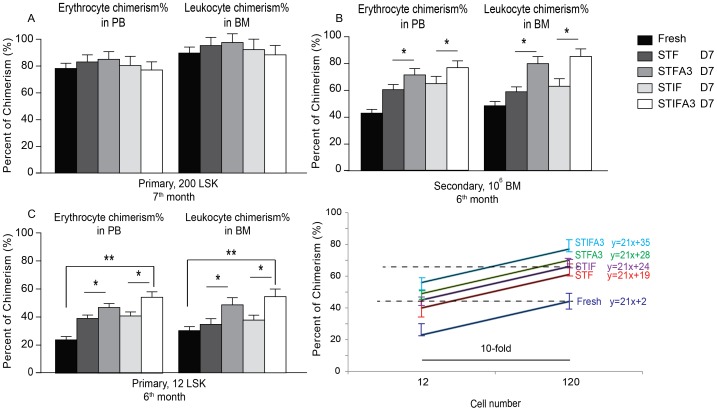
Angptl3 augments the expansion of LT-HSCs from LSK cells. (**A**) Two hundred LSK cells (equivalent to day 0), either fresh or cultured for 7 days under STF, STFA3, STIF, or STIFA3 conditions, were transplanted into sub lethally irradiated recipients. The percent of erythrocyte chimerism in PB and leukocyte chimerism in BM was determined 7 months after transplantation. (B) At 7 months post-transplantation, 10^6^ BM cells from primary recipients were transplanted into secondary recipients, and 6 months after re-transplantation, the percentage of erythrocyte in PB and leukocyte chimerism in BM of secondary recipients was determined. (**C**) Six months post- transplantation of 12 LSK cells into primary recipients, the percentage of erythrocyte chimerism in PB and leukocytes chimerism in BM was determined. (**D**) Serial dilution of LSK cells (120 or 12 cells) transplanted into primary recipients. The data represents erythrocytes chimerism of 120 or 12 LSK transplanted cells after 6 months. For all experiments shown, each group is the combined result of 5 mice. Data is presented ± standard deviation (SD), **P≤0.01.

We next investigated whether culturing of HSCs in the presence of Angptl3 would potentiate long-term hematopoiesis *in vivo*. Long-term repopulating ability (LTRA) assays were performed by transplanting sorted male Lin^−^ (3000, 1000 or 300 cells) or LSK cells (200 cells) into sub-lethally irradiated female α-thalassemia mice with or without prior culturing in STF, STFA3, STIF or STIFA3 media for 7 days (Figure 2A, Figure S2). Near full donor engraftment at 7 months following transplantation of LSK cells was identified in the BM and PB compartments of mice regardless of prior incubation conditions (Figure 2A). One million BM cells from these primary transplanted mice were then retransplanted into secondary recipients, and these mice remained healthy for over 6 months without any symptoms of disease. The percentage of erythrocyte chimerism in the peripheral blood of secondary recipients that received uncultured LSK cells was 43%. However, culturing of the LSK cells prior to transplantation in the first recipient using STF or STIF media increased chimerism levels to 60±3% and 65±5% in the secondary transplanted mice, respectively. Incubation with Angptl3-containing media further increased chimerism levels for STFA3 and STIFA3 media to 74±4% and 77±4% respectively (Figure 2B). The percentages of leukocyte chimerism—established by Y-chromosomal QPCR in bone marrow samples—was similar to the pattern of RBC chimerism levels in peripheral blood in these mice. To quantify the differentiation capacity of cultured LSK cells compared to freshly sorted LSK cells, primary recipient mice were transplanted with 12 or 120 LSK donor cells directly or with offspring cells from 12 or 120 LSK cells following a 7 day culture in STF, STFA3, STIF or STIFA3 media. Transplanting 12 freshly-isolated LSK cells let to a 25±4% donor erythrocyte chimerism level in the peripheral blood 6 months after transplantation (Figure 2C). Culturing of 12 LSK cells in STF or STIF media prior to primary transplantation resulted in donor erythrocyte chimerism of 37±5% or 40±4% in secondary transplanted mice, respectively. Culturing 12 LSK cells in STFA3 or STIFA3 media reconstituted 48±4 and 56±5% of donor erythrocyte chimerism levels, respectively. Again, leukocyte chimerism in the bone marrow phenocopied erythrocyte chimerism levels in peripheral blood of primary transplanted mice. Based on the results from the serial dilution transplantation experiment, culturing LSK cells in STF or STIF media prior to transplantation enhanced ∼10 or ∼6-fold long-term repopulation activity of HSCs. Culturing LSK cells in presence of Angptl3 enhanced number of LT-HSC ∼3-fold, therefore STFA3 and STIFA3 media resulted in ∼17- and ∼32-fold increase in long-term repopulation activity of HSCs compared to non-pretreated LSK cells, respectively (Figure 2D). For Lin^−^ ells, culturing in STF media significantly increased erythrocyte chimerism that was already visible 1 month after transplantation, and became more evident 6 months after transplantation. These results also demonstrated that 7 day culture period seems most optimal. Again, addition of Angptl3 further increased erythrocyte chimerism levels as well as leukocyte chimerism levels (Figures S2A and S2B). Based on these limiting dilution transplantation experiments, we conclude that culturing HSCs in STF media for 7 days increased long-term repopulation capacity ∼10-fold, and was increased by >300-fold for STFA3-incubated Lin^−^ cells (Figure S2C).

### The effect of Angptl3 on *ex vivo* expansion and differentiation capacity of Lin^−^ cells is direct

**Figure 3:**
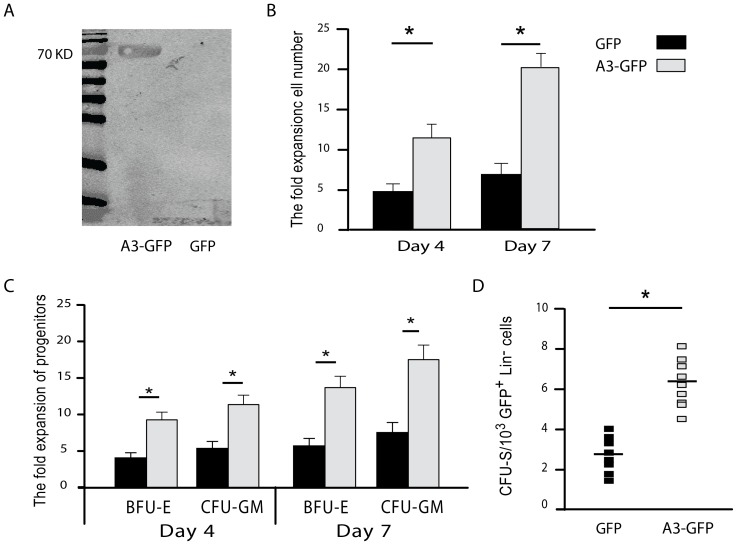
Angptl3 overexpression promotes expansion of HSCs in Lin^−^ cell populations. (**A**) Western blotting analysis for Angptl3 expression in sorted Lin^−^ GFP^+^ cells 2 days following transduction with LV-Angptl3-GFP or the mock control LV-GFP lentiviral particles. (**B**) Total cell fold expansion and (**C**) fold increase in BFU-E or CFU-GM progenitor colonies from transduced Lin^−^ GFP^+^ cells as described under (A) that were cultured for 4 or 7 days. Results are shown relative to day 0, transduced sorted Lin^−^ cells. Mean values are obtained from 5 independent experiments. (**D**) Short-term colony forming unit assay (CFU- S). Lin^−^ GFP^+^ cells were sorted 2 days post transduction and immediately transplanted into lethally irradiated mice. Total numbers of CFU-S colonies in the spleen were counted after 12 days following transplantation (N = 10 mice). *P≤0.05.

To further show that Angptl3 promotes expansion and reconstitution of HSCs, Angptl3 was ectopically expressed in Lin^−^ hematopoietic progenitors by a lentiviral vector with GFP (LV-Angptl3-GFP). As a control, Lin^−^ cells were transduced with a control vector solely expressing GFP (LV-GFP). Western blotting showed that sorted cells expressed the Angptl3 protein 2 days after transduction with LV-Angptl3-GFP whereas the control cells remained negative (Figure 3A). Sorted, transduced cells were then cultured in STIF media and cell numbers were counted after 4 and 7 days. Expression of Angptl3 resulted in a significant increase in cell numbers (Figure 3B), and preservation of progenitor cells and ST-HSCs compared to control cells as assessed by BFU-E and CFU-GM assays (Figure 3C) or CFU-S assay (Figure 3D), respectively.

**Figure 4:**
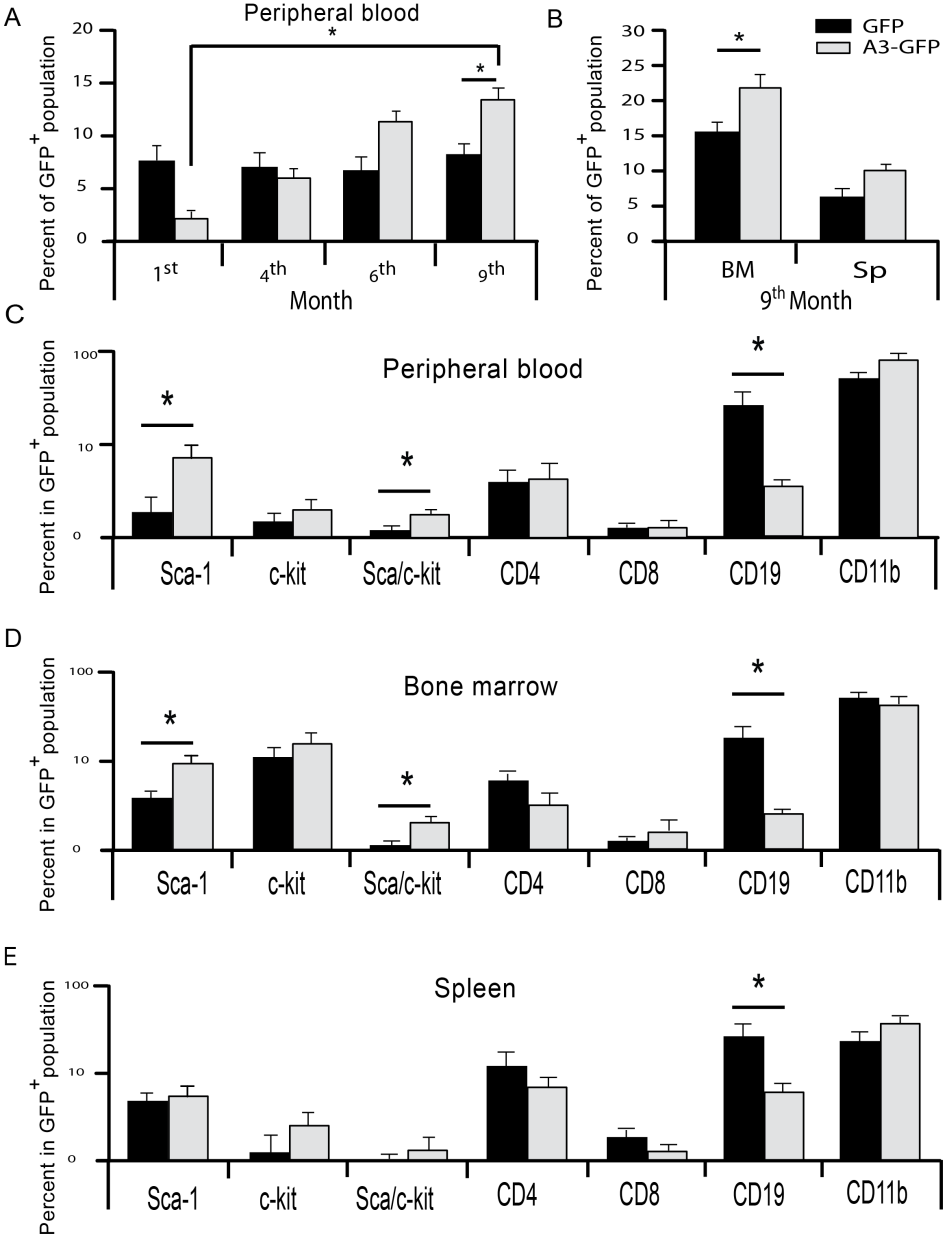
Angptl3 overexpression stimulates the expansion of LT-HSCs in Lin^−^ cell populations *in vivo*. (**A**) Ten thousand sorted BM Lin^−^ Angptl3-GFP^+^ or BM Lin^−^ GFP^+^ cells were transplanted into sub lethally irradiated recipients. The percentage of donor-derived cells (GFP^+^) was determined at 1, 4, 6, and 9 month(s) after transplantation in PB. N = 5 mice per group. The error bars indicate the standard deviation (SD). (**B**) The percentage of GFP positive cells was determined, 9 months after re-transplantation in BM, and spleen in an average of 5 mice per group. (**C**) Nine months post transplantation, the percentage of different blood lineages (Sca-1, c-kit, Sca-1/c-kit, CD4, CD8, CD19, and CD11b cells) in donor-derived cells (GFP^+^) in PB, (**D**) in BM, and (**E**) in spleen was measured by flow cytometry. *P≤0.05.

We then assessed the reconstitution capacity of long-term hematopoiesis for Lin^−^ cells using long- term repopulation ability assays (LTRA). For this, 10,000 sorted, male Lin^−^ GFP^+^ cells were transduced with the control LV-GFP or LV-Angptl3-GFP vector and transplanted into female α-thalassemia recipient mice. All mice remained healthy for over 9 months, and none of these mice exhibited tumor growth or elevated WBC counts. One month post-transplantation, we detected less than 2% GFP^+^cells in the peripheral blood of mice that were transplanted with LV-Angptl3-GFP-transduced Lin^−^ cells when compared to ∼8% in mice transplanted with the control LV-GFP-transduced Lin^−^ cells (Figure 4A). Over the following 8 months, the percentage of GFP^+^ cells in the LV-GFP control group remained stable and varied between 6.5–8%. The percentage of GFP^+^ cells in recipients that were transplanted with LV- Angptl3-GFP-transduced Lin^−^ cells increased over months to 13±2% nine months after transplantation. Percentages of GFP^+^ cells were also significantly elevated in the bone marrow of these mice (23±3%) compared to control mice (15±2%) (Figure 4B). Hematopoietic differentiation was also measured in GFP^+^ cells in the peripheral blood, bone marrow and spleen compartments (Figures 4C–D–E). In the BM and PB, the LV-Angptl3-GFP group contained more GFP^+^ cells with Sca-1^+^ and Sca-1^+^/c-kit^+^ stem cell markers than the LV-GFP control group. Thus Angptl3 may preserve the immature state of HSCs *in vivo*. No difference was observed for the percentage of mature CD4^+^ or CD8^+^ T cells or CD11b^+^ myeloid cells within the GFP^+^ population, but the percentage of CD19^+^ B cells was significantly decreased in the LV-Angptl3-GFP group compared to the control group.

**Figure 5:**
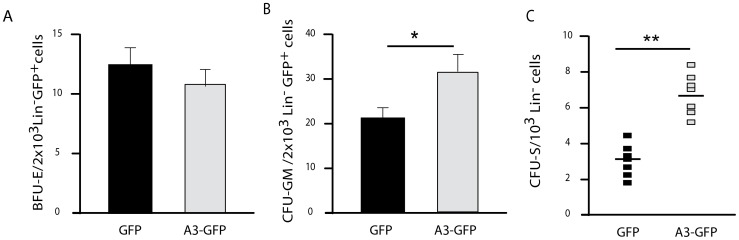
Angptl3 overexpression enhances the expansion of CFU-GM and ST-HSCs *in vivo*. Nine months post transplantation, two thousands sorted BM Lin^−^ GFP^+^ cells of primary recipients (LV-GFP or LV-Angptl3-GFP groups) were plated in a semi-solid colony culture (n = 4). The number of the (**A**) BFU-E and the (**B**) CFU-GM was quantified. The average numbers of colonies from 2×10^3^ plated cells were calculated from quadruplicates. (**C**) One thousand sorted BM Lin^−^ GFP^+^ cells from primary recipients (LV-GFP or LV-Angptl3-GFP groups) were transplanted into lethally irradiated mice (n = 7 mice per group). *P≤0.05, and **P≤0.01.

We next sorted Lin^−^, GFP^+^ cells from BM pools of recipient mice that were transplanted with LV- Angptl3-GFP or LV-GFP-transduced Lin^−^ cells nine months before, and performed progenitor- and short-term colony forming assays. No significant difference in the frequency of BFU-E progenitor cells was identified in the BM Lin^−^, GFP^+^ cell population from both groups (∼12 *vs.* ∼10 BFU-E colonies/2×10^3^ Lin^−^, GFP^+^ cel_ls)_,_ re_sp_e_ct_iv_e_ly_ (Figure 5A_)_. Howe_v_e_r_,_ t_he nu_m_ber of CFU_-_GMs was_ sig_n_ifi_ca_ntl_y_ hig_her for mice transplanted with LV-Angptl3-GFP-transduced Lin^−^ cells relative to the control mice (31±1.6 *vs*. 21±2 CFU-GM colonies/2×103 Lin^−^ GFP^+^) (Figure 5B). In addition, the number of ST-HSCs was 2 fold higher for the LV-Angptl3-GFP group compared to the LV-GFP control group (7±1 vs. 3±1 CFU-S/103 BM Lin^−^, GFP^+^ cells, respectively) (Figure 5C).

## Discussion

Over the past two decades, many attempts have been made to increase the quantity of long-term HSCs by *in vitro* culturing conditions. Although sufficient HSCs are obtained from donors for conventional bone marrow transplantations, expansion of HSC may become more and more relevant for transplantations relying on umbilical cord blood HSCs or transplantation of limiting, genetically-modified HSCs. Serum free expansion cultures of HSCs using SCF, TPO and Flt3L-supplemented media (STF) were shown to maintain the number of murine long term HSCs but led to increased numbers of human primitive hematopoietic progenitors with preserved engraftment potential [9], [37], [38]. Zhang *et al*. reported a new combination of growth factors that included SCF, TPO, IGF-2 and FGF-1 (STIF) that was supplemented with angiopoietin-like protein 2 or 3 (Angptl2, Angptl3) that supported *ex-vivo* expansion of murine long-term HSC frequencies by 24- to 30-fold in 10 days [26], [27]. Furthermore, IGF- binding protein 2 (IGFBP2) and Angptl5 (A5) were introduced as additional factors that support human HSC expansion [39]. Using SCF [40], TPO [41], and FGF-1 supplemented with IGFBP2 and Angptl5, the number of human stem cells that can repopulate NOD-SCID mice increased ∼20-fold compared to non- cultured HSCs [42].

In recent years, other factors have been identified that support *in vitro* expansion of HSCs. These stimulate the Wnt [43] and Notch [44] pathways that have been implicated in the regulation of HSCs fate [45]. Wnt signaling may inhibit glycogen synthase kinase 3 (GSK-3) thereby stabilizing β-actin that supports expansion of HSCs. However, inhibition of GSK-3 also results in the upregulation of the mammalian target of rapamycin (mTOR), which promotes the proliferation of committed progenitor cells. It was shown that a dual inhibitor for both GSK-3 and mTOR resulted in maintenance and expansion of HSCs *in vitro*, even in the absence of cytokines [45]. Microenvironmental factors such as pleiotrophin may also enhance HSC expansion *in vitro* and improved HSC repopulating capacity by ∼10-fold using competitive transplantation assays [46]. Chemical compounds may have also have an effect: All-trans retinoic acid (ATRA) in combination with SCF, FLT3L, IL-6, and IL-11-enriched medium prolonged the repopulating capacity of HSCs [47]. The Cu^2+^-chelator tetraethylenepentamine (TEPA) enhanced *ex vivo* expansion of CD34^+^CD38^−^ and CD34+ Lin^−^ subsets isolated from umbilical cord blood samples, as well enhanced their short-term repopulating activity in NOD-SCID mice [48]. The histon deacetylase inhibitor (HDI) valproic acid [49] and StemRegenin1—a small molecule antagonist of the Aryl hydrocarbon receptor [50]—were both able to promote long-term hematopoiesis following transplantation of cultured HSCs. Prostaglandin E2 (PGE2) may also be useful for *ex vivo* expansion of HSC [51], [52]. Taken together, several factors can be utilized to optimize most prominent *ex vivo* expansion conditions for HSCs.

The optimal mix of cytokines and culture conditions that warrant most optimal *ex vivo* HSC expansion is not yet clear. We therefore explored *ex vivo* expansion of HSCs using two different combinations of growth factors (STF and STIF) with or without the addition of Angptl3. Angptl3 may provide optimal preservation of stemness and promote long-term hematopoiesis without provoking leukemogenic or toxic effects following transplantation. The Angptl3 polypeptide (455 amino acids) has all the characteristic features of angiopoietins, and includes a signal peptide, an extended helical domain that forms dimeric or trimeric coiled coils, a short linker peptide and a globular fibrinogen homology domain (FHD). Angptl3 is expressed by BM-endothelial and other stromal cells, and binds directly to the cell-surface on HSCs [27]. For this, HSCs have the immune-inhibitory receptor human leukocyte immunoglobulin-like receptor B2 (LILRB2) that can bind various ANGPTLs. In mice, the orthologue paired immunoglobulin-like receptor (PIRB) has also been identified as a receptor for various Angptl proteins [28]. Binding of Angptl3 to the receptors of HSCs provoke differences in the expression of cell cycle regulators and transcription factors like repression of Ikaros but upregulation of Hes1 and Hoxa9, which are both important regulators for HSC self-renewal and differentiation [27]. Angptl3-null mice were shown to have a 3-fold higher level of Ikaros [27]. Ectopic expression of Ikaros in mouse HSCs severely reduced hematopoietic reconstitution capacity following transplantation [27], which suggests that Angptl3 may be one of the most important regulators for HSC stemness [27].

Incubation of HSCs in STF and STIF media has a similar effect on the expansion of overall cell numbers as well as progenitors and short-term HSC numbers. We now demonstrate that STIF is superior over STF to preserve the total number of LSK cells, resulting in improved long-term hematopoietic repopulation results following transplantation. The addition of Angptl3 does not change the overall expansion rate, but improves the preservation of HSC stemness that support short-term and long-term hematopoiesis. Culturing LSK cells in the presence of STFA3 for 7 days improves short-term hematopoiesis in CFU-S assays by ∼40-fold compared to the control group. The long-term repopulating capacity of LSK cells is enhanced ∼32-fold following a 7 days incubation in STIFA3 media as estimated from peripheral blood erythrocyte chimerism levels. The effect of Angptl3 is clear both on Lin^−^ cells as well as on highly purified HSCs (LSK-cells), indicating that the effect of Angptl3 is directly on HSCs and preserves stemness. In addition to a direct effect of Angptl3 on stem cells, we observed that the repopulating capacity of long-term hematopoiesis after transplantation was better for Lin^−^ cells than for highly purified LSK cells, implying that Angptl3 may also exert an additional effect on accessory cells that support the maintenance of HSCs.

The effect of Angptl3 on preserving stemness of HSCs was further supported by finding reduced numbers of circulating CD19^+^ B-cells following transplantation of Angptl3-expressing HSCs. As overexpression of Angptl3 down-regulates Ikaros in HSCs [27], and since Ikaros deletions are frequently observed in B-cell acute lymphoblastic leukemias [53], this may provide an alternative explanation for the reduced circulating mature B-cells following transplantation of Angptl3-overexpressing HSCs. In our experiments, we did not find any evidence for leukemia or other types of cancer or toxicity at nine months following transplantation of Angptl3-overexpressing donor HSCs.

## Conclusions

To conclude, we showed that the combination of five growth factors [SCF, TPO, IGF-2, FGF-1 and Angptl3 (STIFA3)] yielded a very significant expansion of stem cells that were able to provide long-term hematopoiesis in mice. This combination may promote superior engraftment over existing methods when using minimal stem cell numbers in the case of genetically-modified HSCs to treat genetic diseases or transplants that rely on umbilical cord blood stem cell donors.

## Supporting Information

Figure S1 Angptl3 promotes the expansion of HSCs in Lin^−^ cell populations. Lin^−^ cells were cultured in STF, STFA3, STIF, or STIFA3 medium for 10 days. (**A**) The mean fold increase in total cell numbers was measured relative to day 0. The results of five independent experiments are shown. The error bars indicate the standard deviation (SD). * signifies P<0.05. (**B, C**) Colony forming-units (BFU-E and CFU-GM) of Lin^−^ cells cultured for 4, 7 or 10 days relative to day 0. The results of 5 independent experiments in duplicates are shown. (**D**) The CFU-S (12-day) expansion of Lin^−^ cells cultured for 4 or 7 days in the presence of 4 distinct combinations of growth factors relative to day 0. The results of two independent experiments are shown. N = 7 mice per group.
(TIF)

Figure S2 Angptl3 stimulates the expansion of LT-HSCs in Lin^−^ cell populations. (**A**) One thousand Lin^−^ cells (equivalent to day 0), either fresh or cultured for 4, 7, or 10 days under STF or STFA3 conditions were transplanted into sub lethally irradiated recipients. The percentage of erythrocyte chimerism was determined 1 month and 6 months after transplantation. Five mice were used per group. The error bars indicate the standard deviation (SD). P-values equal or lower that p = 0.05 are marked by an asterisks. (**B**) The percentage of leukocytes chimerism of transplantation of 1000 Lin^−^ cells (equivalent to day 0), either fresh or cultured for 7 days under STF or STFA3 conditions was determined in bone marrow of recipients, 6 months after retransplantation. N = 5 mice per group. (**C**) Serial dilution of fresh Lin^−^ cells (3000 or 1000) or cultured cells (1000 or 300) for 7 days were transplanted into primary recipients. The data represents erythrocyte chimerism of 3000, 1000, or 300 transplanted Lin^−^ cells after 6 months. N = 5 mice per group. The error bars indicate the standard deviation.
(TIF)

Table S1 Angptl3 promotes the expansion of HSCs in Lin^−^ cell populations, primary data. (**A, B**) Two thousands Lin^−^ cells fresh or cultured in STF, STFA3, STIF, or STIFA3 medium were plated in 35 mm culture dishes that contained 1 ml of enriched DMEM culture medium that was supplemented with 0.8% (wt/vol) methylcellulose. 2 weeks post plating, colonies were counted in each dish. The experiments were performed in duplicates. The result of colony forming-units (BFU-E and CFU-GM) of Lin^−^ cells cultured for 4, 7 or 10 is presented as a mean of duplicates. The results of five independent experiments are shown. (**C**) The CFU-S (12-day) colony numbers of uncultured Lin^−^ cells (3000, 1000 cells) or cultured for 4 or 7 days (1000, 100) in the presence of 4 distinct combinations of growth factors. The results of two serial dilution are shown. N = 7 mice per group.
(TIF)

Table S2 Angptl3 promotes the expansion of |HSCs in LSK cell populations, primary data. Two hundred LSK cells were cultured in STF, STFA3, STIF, or STIFA3 medium for 7 days were plated in 35 mm culture dishes that contained 1 ml of enriched DMEM culture medium that was supplemented with 0.8% (wt/vol) methylcellulose. 2 weeks post plating, colonies were counted in each dish. The experiments were performed in duplicates. The results of 5 independent experiments are shown The result of colony forming-units (BFU-E and CFU-GM) of LSK cells cultured 7 days is presented as a mean of duplicates. (**A**) Colony forming-units of BFU-E and (**B**) CFU-GM. (**D**) The CFU-S (12-day) colony numbers of transplanting 100 LSK cells fresh or cultured for 7 days in the presence of 4 distinct combinations of growth factors 12 days post transplantation into lethally irradiated mice. The results of two independent experiments are shown. N = 6 mice per group.
(TIF)

## References

[1] Attar EC, Scadden DT (2004) Regulation of hematopoietic stem cell growth. Leukemia 18: 1760–1768. 15457182 10.1038/sj.leu.2403515

[2] Reya T (2003) Regulation of Hematopoietic Stem Cell Self-Renewal. Recent Prog Horm Res 58: 283–295. 12795424 10.1210/rp.58.1.283

[3] Boyiadzis M, Pavletic S (2004) Haematopoietic stem cell transplantation: indications, clinical developments and future directions. Expert Opinion on Pharmacotherapy 5: 97–108. 14680439 10.1517/14656566.5.1.97

[4] Gratwohl A, Passweg J, Bocelli-Tyndall C, Fassas A, van Laar JM, et al (2005) Autologous hematopoietic stem cell transplantation for autoimmune diseases. Bone Marrow Transplant 35: 869–879. 15765114 10.1038/sj.bmt.1704892

[5] Samantha L, Ginn JAC, Kramer B, Smyth CM, Wong M, et al (2005) Treatment of an infant with X-linked severe combined immunodeficiency (SCID-X1) by gene therapy in Australia. MJA 182 (9): 458–463. 10.5694/j.1326-5377.2005.tb06785.x 15865589

[6] Shizuru JA, Negrin RS, Weissman IL (2005) Hematopoietic Stem and Progenitor Cells: Clinical and Preclinical Regeneration of the Hematolymphoid System. Annual Review of Medicine 56: 509–538. 10.1146/annurev.med.54.101601.152334 15660525

[7] Sauvageau G, Iscove NN, Humphries RK (2004) In vitro and in vivo expansion of hematopoietic stem cells. Oncogene 23: 7223–7232. 15378082 10.1038/sj.onc.1207942

[8] Sorrentino BP (2004) Clinical strategies for expansion of haematopoietic stem cells. Nat Rev Immunol 4: 878–888. 15516967 10.1038/nri1487

[9] Goff JP, Shields DS, Greenberger JS (1998) Influence of Cytokines on the Growth Kinetics and Immunophenotype of Daughter Cells Resulting From the First Division of Single CD34+Thy-1+lin− Cells. Blood 92: 4098–4107. 9834215

[10] Antonchuk J, Sauvageau G, Humphries RK (2002) HOXB4-induced expansion of adult hematopoietic stem cells ex vivo. Cell 109: 39. 11955445 10.1016/s0092-8674(02)00697-9

[11] Sauvageau G, Thorsteinsdottir U, Eaves CJ (1995) Overexpression of HOXB4 in hematopoietic cells causes the selective expansion of more primitive populations in vitro and in vivo. Genes Dev 9: 1753. 7622039 10.1101/gad.9.14.1753

[12] Hacein-Bey-Abina S, Von Kalle C, Schmidt M, McCormack MP, Wulffraat N, et al (2003) LMO2-Associated Clonal T Cell Proliferation in Two Patients after Gene Therapy for SCID-X1. Science 302: 415–419. 14564000 10.1126/science.1088547

[13] Matthews JM, Lester K, Joseph S, Curtis DJ (2013) LIM-domain-only proteins in cancer. Nat Rev Cancer 13: 111–122. 23303138 10.1038/nrc3418

[14] McCormack MP, Rabbitts TH (2004) Activation of the T-Cell Oncogene LMO2 after Gene Therapy for X-Linked Severe Combined Immunodeficiency. New England Journal of Medicine 350: 913–922. 14985489 10.1056/NEJMra032207

[15] Pike-Overzet K, de Ridder D, Weerkamp F, Baert MR, Verstegen MM, et al (2007) Ectopic retroviral expression of LMO2, but not IL2Rgamma, blocks human T-cell development from CD34+ cells: implications for leukemogenesis in gene therapy. Leukemia 21: 754–763. 17268520 10.1038/sj.leu.2404563

[16] Stier S, Cheng T, Dombkowski D, Carlesso N, Scadden DT (2002) Notch1 activation increases hematopoietic stem cell self-renewal in vivo and favors lymphoid over myeloid lineage outcome. Blood 99: 2369–2378. 11895769 10.1182/blood.v99.7.2369

[17] Linnekin D, Keller JR, Ferris DK, Mou SM, Broudy V, et al (1995) Stem cell factor induces phosphorylation of a 200 kDa protein which associates with c-kit. Growth Factors 12: 57–67. 8527164 10.3109/08977199509003214

[18] Mouthon MA, Van der Meeren A, Gaugler MH, Visser TP, Squiban C, et al (1999) Thrombopoietin promotes hematopoietic recovery and survival after high-dose whole body irradiation. Int J Radiat Oncol Biol Phys 43: 867–875. 10098443 10.1016/s0360-3016(98)00477-5

[19] Yoshihara H, Arai F, Hosokawa K, Hagiwara T, Takubo K, et al (2007) Thrombopoietin/MPL Signaling Regulates Hematopoietic Stem Cell Quiescence and Interaction with the Osteoblastic Niche. Cell Stem Cell 1: 685–697. 18371409 10.1016/j.stem.2007.10.020

[20] Lisovsky M, Estrov Z, Zhang X, Consoli U, Sanchez-Williams G, et al.. (1996) Flt3 ligand stimulates proliferation and inhibits apoptosis of acute myeloid leukemia cells: regulation of Bcl-2 and Bax. pp. 3987–3997. 8916965

[21] Yeoh JSG, van Os R, Weersing E, Ausema A, Dontje B, et al.. (2006) Fibroblast Growth Factor-1 and -2 Preserve Long-Term Repopulating Ability of Hematopoietic Stem Cells in Serum-Free Cultures. pp. 1564–1572. 10.1634/stemcells.2005-0439 16527900

[22] de Haan G, Weersing E, Dontje B, van Os R, Bystrykh LV, et al (2003) In Vitro Generation of Long-Term Repopulating Hematopoietic Stem Cells by Fibroblast Growth Factor-1. Developmental Cell 4: 241–251. 12586067 10.1016/s1534-5807(03)00018-2

[23] Reya T, Duncan AW, Ailles L, Domen J, Scherer DC, et al (2003) A role for Wnt signalling in self-renewal of haematopoietic stem cells. Nature 423: 409–414. 12717450 10.1038/nature01593

[24] Chou S, Lodish HF (2010) Fetal liver hepatic progenitors are supportive stromal cells for hematopoietic stem cells. Proc Nat Aca Sciences 107: 7799–7804. 10.1073/pnas.1003586107 PMC2867886 20385801

[25] Zhang CC, Lodish HF (2004) Insulin-like growth factor 2 expressed in a novel fetal liver cell population is a growth factor for hematopoietic stem cells. Blood 103: 2513–2521. 14592820 10.1182/blood-2003-08-2955

[26] Zhang CC, Kaba M, Ge G, Xie K, Tong W, et al (2006) Angiopoietin-like proteins stimulate ex vivo expansion of hematopoietic stem cells. Nat Med 12: 240–245. 16429146 10.1038/nm1342 PMC2771412

[27] Zheng J, Huynh H, Umikawa M, Silvany R, Zhang CC (2011) Angiopoietin-like protein 3 supports the activity of hematopoietic stem cells in the bone marrow niche. Blood 117: 470–479. 20959605 10.1182/blood-2010-06-291716 PMC3031476

[28] Zheng J, Umikawa M, Cui C, Li J, Chen X, et al (2012) Inhibitory receptors bind ANGPTLs and support blood stem cells and leukaemia development. Nature 485: 656–660. 22660330 10.1038/nature11095 PMC3367397

[29] Broxmeyer HE, Srour EF, Cooper S, Wallace CT, Hangoc G, et al (2012) Angiopoietin-like-2 and -3 act through their coiled-coil domains to enhance survival and replating capacity of human cord blood hematopoietic progenitors. Blood Cells, Molecules, and Diseases 48: 25–29. 10.1016/j.bcmd.2011.09.004 PMC3249507 21983347

[30] Amendola M, Venneri MA, Biffi A, Vigna E, Naldini L (2005) Coordinate dual-gene transgenesis by lentiviral vectors carrying synthetic bidirectional promoters. Nat Biotech 23: 108–116. 10.1038/nbt1049 15619618

[31] Dull T, Zufferey R, Kelly M, Mandel RJ, Nguyen M, et al (1998) A Third-Generation Lentivirus Vector with a Conditional Packaging System. Journal of Virology 72: 8463–8471. 9765382 10.1128/jvi.72.11.8463-8471.1998 PMC110254

[32] Zufferey R, Dull T, Mandel RJ, Bukovsky A, Quiroz D, et al (1998) Self-Inactivating Lentivirus Vector for Safe and Efficient In Vivo Gene Delivery. Journal of Virology 72: 9873–9880. 9811723 10.1128/jvi.72.12.9873-9880.1998 PMC110499

[33] Merchav S, Wagemaker G (1984) Detection of murine bone marrow granulocyte/macrophage progenitor cells (GM-CFU) in serum-free cultures stimulated with purified M-CSF or GM-CSF. Int J Cell Cloning 2: 356–367. 6335156 10.1002/stem.5530020604

[34] Wagemaker G, Visser TP (1980) Erythropoietin-independent regeneration of erythroid progenitor cells following multiple injections of hydroxyurea. Cell Tissue Kinet 13: 505–517. 7459981 10.1111/j.1365-2184.1980.tb00491.x

[35] Guilbert LJ, Iscove NN (1976) Partial replacement of serum by selenite, transferrin, albumin and lecithin in haemopoietic cell cultures. Nature 263: 594–595. 1086432 10.1038/263594a0

[36] van den Bos C, van Gils FC, Bartstra RW, Wagemaker G (1992) Flow cytometric analysis of peripheral blood erythrocyte chimerism in alpha-thalassemic mice. Cytometry 13: 659–662. 1451598 10.1002/cyto.990130616

[37] Wognum AW, Visser TP, Peters K, Bierhuizen MF, Wagemaker G (2000) Stimulation of mouse bone marrow cells with kit ligand, FLT3 ligand, and thrombopoietin leads to efficient retrovirus-mediated gene transfer to stem cells, whereas interleukin 3 and interleukin 11 reduce transduction of short- and long-term repopulating cells. Hum Gene Ther 11: 2129–2141. 11044914 10.1089/104303400750001435

[38] Luens KM, Travis MA, Chen BP, Hill BL, Scollay R, et al (1998) Thrombopoietin, kit Ligand, and flk2/flt3 Ligand Together Induce Increased Numbers of Primitive Hematopoietic Progenitors From Human CD34+Thy-1+Lin− Cells With Preserved Ability to Engraft SCID-hu Bone. Blood 91: 1206–1215. 9454750

[39] Huynh H, Iizuka S, Kaba M, Kirak O, Zheng J, et al (2008) Insulin-Like Growth Factor-Binding Protein 2 Secreted by a Tumorigenic Cell Line Supports Ex Vivo Expansion of Mouse Hematopoietic Stem Cells. Stem Cells 26: 1628–1635. 18369099 10.1634/stemcells.2008-0064 PMC2678056

[40] Li CL, Johnson GR (1994) Stem cell factor enhances the survival but not the self-renewal of murine hematopoietic long-term repopulating cells. Blood 84: 408–414. 7517713

[41] Broudy VC, Lin NL, Kaushansky K (1995) Thrombopoietin (c-mpl ligand) acts synergistically with erythropoietin, stem cell factor, and interleukin-11 to enhance murine megakaryocyte colony growth and increases megakaryocyte ploidy in vitro. Blood 85: 1719–1726. 7535585

[42] Zhang CC (2008) Angiopoietin-like 5 and IGFBP2 stimulate ex vivo expansion of human cord blood hematopoietic stem cells as assayed by NOD/SCID transplantation. Blood 111: 3415–3423. 18202223 10.1182/blood-2007-11-122119 PMC2275010

[43] Willert K, Brown JD, Danenberg E (2003) Wnt proteins are lipid-modified and can act as stem cell growth factors. Nature 423: 448. 12717451 10.1038/nature01611

[44] Varnum-Finney B, Brashem-Stein C, Bernstein ID (2003) Combined effects of Notch signaling and cytokines induce a multiple log increase in precursors with lymphoid and myeloid reconstituting ability. Blood 101: 1784–1789. 12411302 10.1182/blood-2002-06-1862

[45] Masuda S, Li M, Izpisua Belmonte JC (2013) Niche-less maintenance of HSCs by 2i. Cell Res 23: 458–459. 23357848 10.1038/cr.2013.14 PMC3616430

[46] Himburg HA, Muramoto GG, Daher P, Meadows SK, Russell JL, et al (2010) Pleiotrophin regulates the expansion and regeneration of hematopoietic stem cells. Nat Med 16: 475–482. 20305662 10.1038/nm.2119 PMC3689427

[47] Purton LE, Bernstein ID, Collins SJ (2000) All-trans retinoic acid enhances the long-term repopulating activity of cultured hematopoietic stem cells. Blood 95: 470–477. 10627451

[48] Peled T, Landau E, Mandel J, Glukhman E, Goudsmid NR, et al (2004) Linear polyamine copper chelator tetraethylenepentamine augments long-term ex vivo expansion of cord blood-derived CD34+ cells and increases their engraftment potential in NOD/SCID mice. Experimental Hematology 32: 547–555. 15183895 10.1016/j.exphem.2004.03.002

[49] Bug G, Gül H, Schwarz K, Pfeifer H, Kampfmann M, et al (2005) Valproic Acid Stimulates Proliferation and Self-renewal of Hematopoietic Stem Cells. Cancer Research 65: 2537–2541. 15805245 10.1158/0008-5472.CAN-04-3011

[50] Boitano AE, Wang J, Romeo R, Bouchez LC, Parker AE, et al (2010) Aryl Hydrocarbon Receptor Antagonists Promote the Expansion of Human Hematopoietic Stem Cells. Science 329: 1345–1348. 20688981 10.1126/science.1191536 PMC3033342

[51] Ikushima YM, Arai F, Hosokawa K, Toyama H, Takubo K, et al (2013) Prostaglandin E2 regulates murine hematopoietic stem/progenitor cells directly via EP4 receptor and indirectly through mesenchymal progenitor cells. Blood 121: 1995–2007. 23315170 10.1182/blood-2012-06-437889

[52] North TE, Goessling W, Walkley CR, Lengerke C, Kopani KR, et al (2007) Prostaglandin E2 regulates vertebrate haematopoietic stem cell homeostasis. Nature 447: 1007–1011. 17581586 10.1038/nature05883 PMC2775137

[53] Mullighan CG, Su X, Zhang J, Radtke I, Phillips LAA, et al (2009) Deletion of IKZF1 and Prognosis in Acute Lymphoblastic Leukemia. New England Journal of Medicine 360: 470–480. 19129520 10.1056/NEJMoa0808253 PMC2674612

