# Supplementary figures and images for: Angiopoietin-Like Protein 3 Promotes Preservation of Stemness during Ex Vivo Expansion of Murine Hematopoietic Stem Cells
Source: PLoS One. 2014 Aug 29;9(8):e105642. doi: 10.1371/journal.pone.0105642 (PMC4149469; doi:10.1371/journal.pone.0105642)

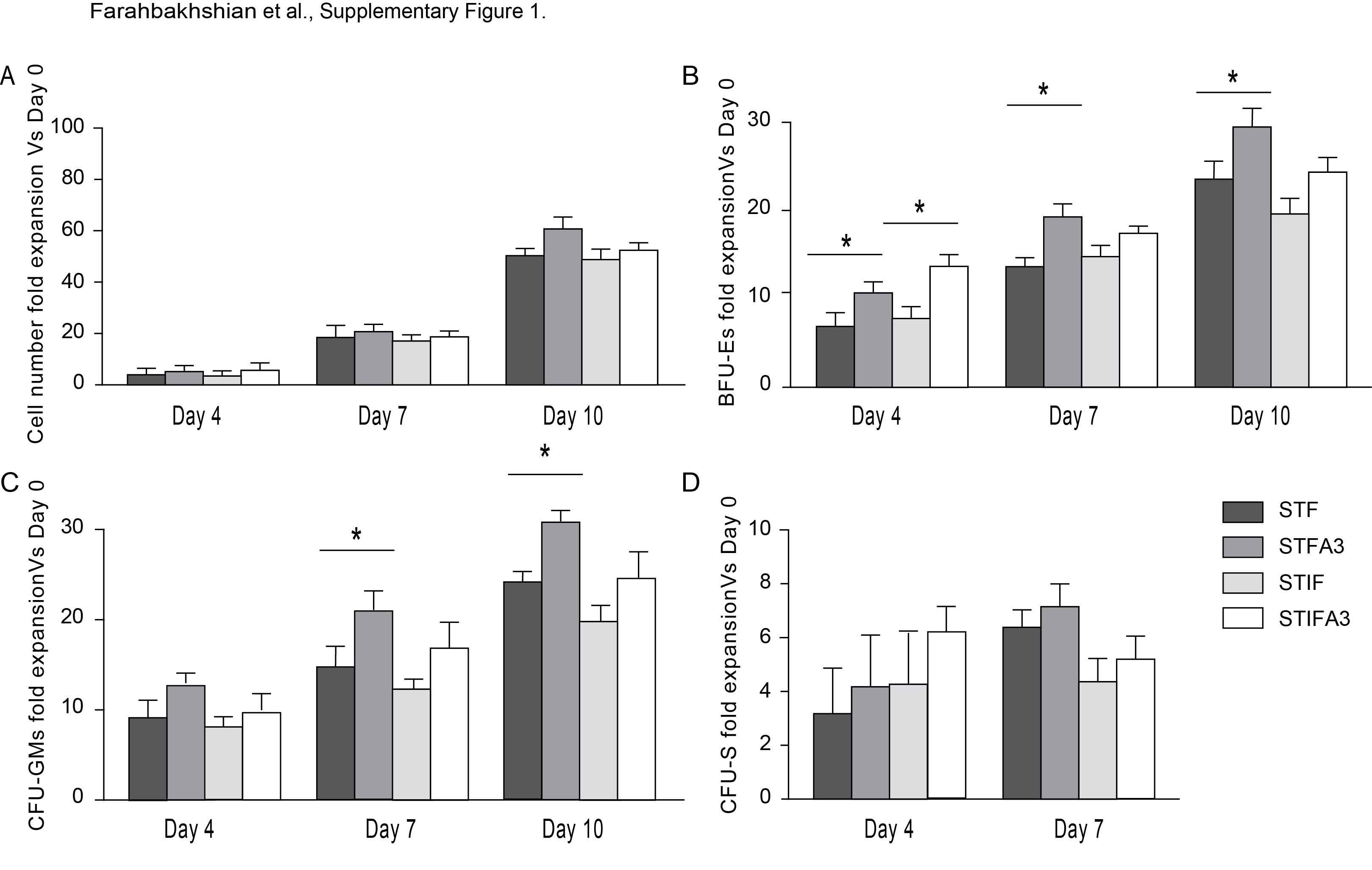

Supplement: Figure S1 — Angptl3 promotes the expansion of HSCs in Lin− cell populations. Lin− cells were cultured in STF, STFA3, STIF, or STIFA3 medium for 10 days. (A) The mean fold increase in total cell numbers was measured relative to day 0. The results of five independent experiments are shown. The error bars indicate the standard deviation (SD). * signifies P<0.05. (B, C) Colony forming-units (BFU-E and CFU-GM) of Lin− cells cultured for 4, 7 or 10 days relative to day 0. The results of 5 independent experiments in duplicates are shown. (D) The CFU-S (12-day) expansion of Lin− cells cultured for 4 or 7 days in the presence of 4 distinct combinations of growth factors relative to day 0. The results of two independent experiments are shown. N = 7 mice per group. (TIF) [file pone.0105642.s001.tif]

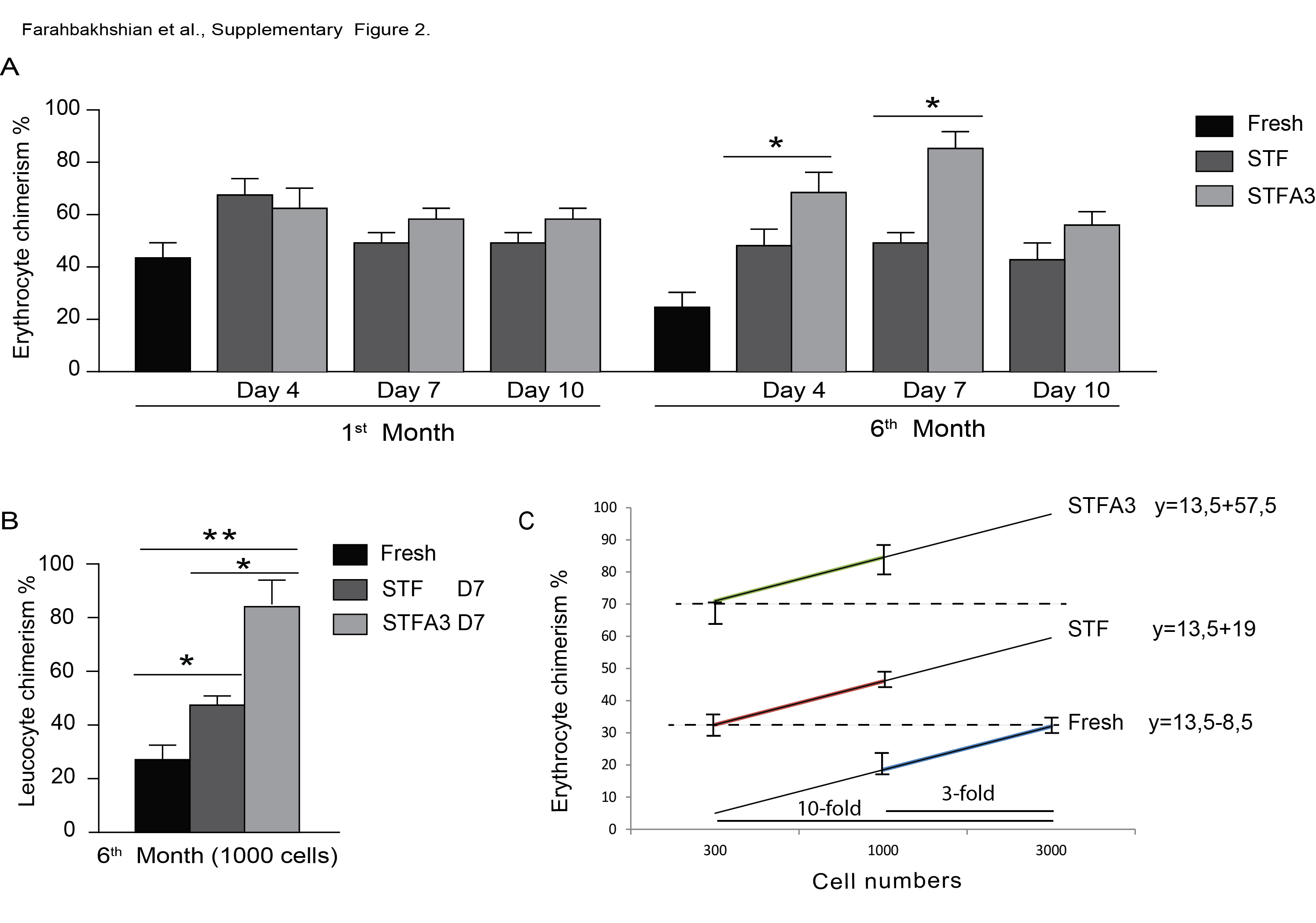

Supplement: Figure S2 — Angptl3 stimulates the expansion of LT-HSCs in Lin− cell populations. (A) One thousand Lin− cells (equivalent to day 0), either fresh or cultured for 4, 7, or 10 days under STF or STFA3 conditions were transplanted into sub lethally irradiated recipients. The percentage of erythrocyte chimerism was determined 1 month and 6 months after transplantation. Five mice were used per group. The error bars indicate the standard deviation (SD). P-values equal or lower that p = 0.05 are marked by an asterisks. (B) The percentage of leukocytes chimerism of transplantation of 1000 Lin− cells (equivalent to day 0), either fresh or cultured for 7 days under STF or STFA3 conditions was determined in bone marrow of recipients, 6 months after retransplantation. N = 5 mice per group. (C) Serial dilution of fresh Lin− cells (3000 or 1000) or cultured cells (1000 or 300) for 7 days were transplanted into primary recipients. The data represents erythrocyte chimerism of 3000, 1000, or 300 transplanted Lin− cells after 6 months. N = 5 mice per group. The error bars indicate the standard deviation. (TIF) [file pone.0105642.s002.tif]

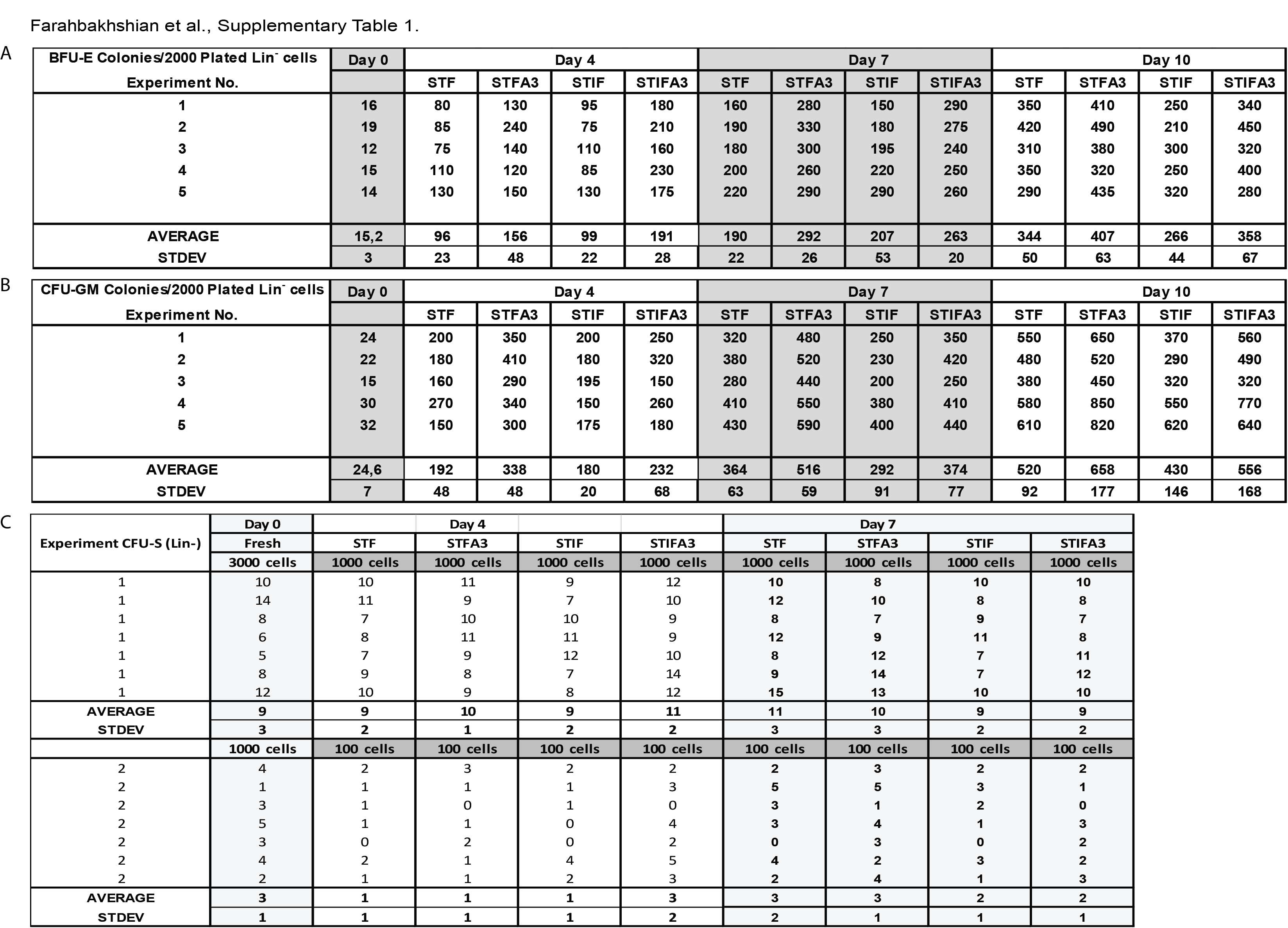

Supplement: Table S1 — Angptl3 promotes the expansion of HSCs in Lin− cell populations, primary data. (A, B) Two thousands Lin− cells fresh or cultured in STF, STFA3, STIF, or STIFA3 medium were plated in 35 mm culture dishes that contained 1 ml of enriched DMEM culture medium that was supplemented with 0.8% (wt/vol) methylcellulose. 2 weeks post plating, colonies were counted in each dish. The experiments were performed in duplicates. The result of colony forming-units (BFU-E and CFU-GM) of Lin− cells cultured for 4, 7 or 10 is presented as a mean of duplicates. The results of five independent experiments are shown. (C) The CFU-S (12-day) colony numbers of uncultured Lin− cells (3000, 1000 cells) or cultured for 4 or 7 days (1000, 100) in the presence of 4 distinct combinations of growth factors. The results of two serial dilution are shown. N = 7 mice per group. (TIF) [file pone.0105642.s003.tif]

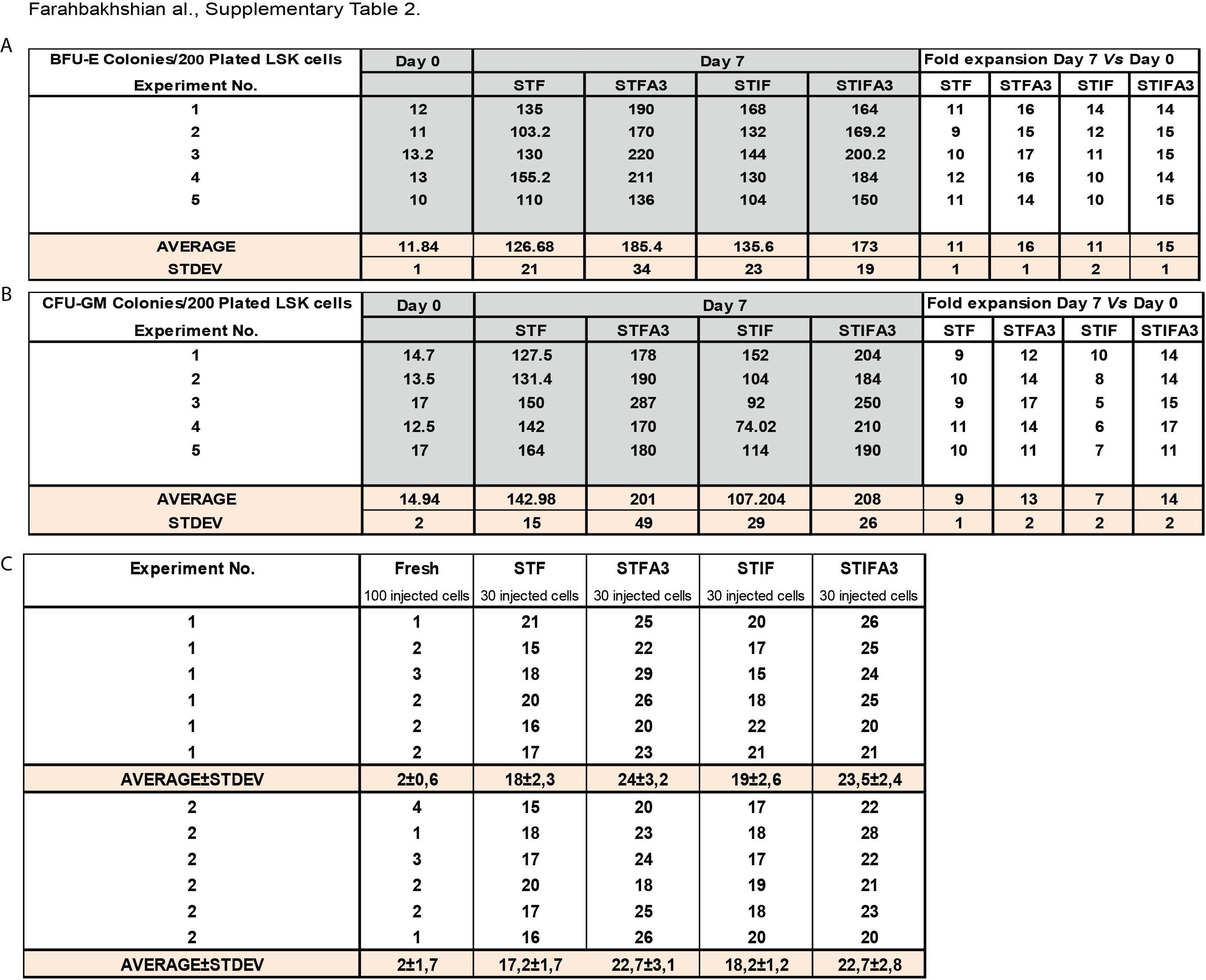

Supplement: Table S2 — Angptl3 promotes the expansion of |HSCs in LSK cell populations, primary data. Two hundred LSK cells were cultured in STF, STFA3, STIF, or STIFA3 medium for 7 days were plated in 35 mm culture dishes that contained 1 ml of enriched DMEM culture medium that was supplemented with 0.8% (wt/vol) methylcellulose. 2 weeks post plating, colonies were counted in each dish. The experiments were performed in duplicates. The results of 5 independent experiments are shown The result of colony forming-units (BFU-E and CFU-GM) of LSK cells cultured 7 days is presented as a mean of duplicates. (A) Colony forming-units of BFU-E and (B) CFU-GM. (D) The CFU-S (12-day) colony numbers of transplanting 100 LSK cells fresh or cultured for 7 days in the presence of 4 distinct combinations of growth factors 12 days post transplantation into lethally irradiated mice. The results of two independent experiments are shown. N = 6 mice per group. (TIF) [file pone.0105642.s004.tif]
